# Environmental, social, and behavioral challenges of the human circadian clock in real-life conditions

**DOI:** 10.3389/fphys.2024.1347377

**Published:** 2024-03-07

**Authors:** Bettina Tassino, Ana Silva

**Affiliations:** ^1^ Sección Etología, Instituto de Biología, Facultad de Ciencias, Universidad de la República, Montevideo, Uruguay; ^2^ Grupo de Investigación en Cronobiología, Universidad de la República, Montevideo, Uruguay; ^3^ Laboratorio de Neurociencias, Instituto de Biología, Facultad de Ciencias, Universidad de la República, Montevideo, Uruguay

**Keywords:** circadian phase, zeitgeber, Antarctica, shift work, light exposure, physical activity

## Abstract

Urban environments, in which ambient light has become a less-reliable entrainer, are challenging for the biological clock to maintain performance. As a consequence, human circadian rhythms are less robust and more variable among individuals. Assessing the individual phase of entrainment, as well as its plastic shifts in response to disturbances of the physical and social environment, is a way to measure circadian disruption. However, this is still difficult to address in real-life scenarios in which several factors modulate the circadian phase not always in a concerted manner. In this perspective, we present the contribution of two real-life situations, in which the circadian system is challenged by important alterations in entraining signals: 1) a trip to the Antarctic summer (socio-environmental challenge), and 2) dancers trained in morning/night shifts (socio-behavioral challenge). Both natural chronobiological experiments are helpful in exploring the functioning and plasticity of the circadian clock and allow for considering individual characteristics and history.

## 1 Introduction

The circadian clock has enabled organisms to anticipate regular daily environmental cycles by using the reliable information of the light-dark cycle to synchronize all body functions to the external 24 h-day. However, the environment in which the biological clock has evolved for millions of years is way far from the urban environment in which most of the human population currently lives. In particular, the light-dark cycle has become a less robust and less reliable synchronizing signal (zeitgeber), while other factors such as food (McHill et al., 2019), social pressures (Crowley et al., 2006; Razavi et al., 2019), exercise (Youngstedt et al., 2019), and stress (Tahara et al., 2017), have been postulated as additional circadian modulators. As a consequence, human circadian rhythms have also become less robust, more variable among individuals, and more desynchronized. Several conditions of the real world have forced circadian rhythms to be decoupled from the 24 h-cycle (Czeisler, 2013), from the solar time (Roenneberg et al., 2019b), and even led to desynchronization between central and peripheral rhythms (Panda and Hogenesch, 2004; Manoogian et al., 2022). The biological clock has proven to display a huge plasticity to maintain performance under challenging environmental and social conditions. However, circadian disruption caused by circadian misalignment is a well-documented health risk factor usually associated with other dysfunctions such as chronic sleep deficit (Vetter, 2020).

The rhythm of melatonin secretion provides the best available estimation of the timing of the internal clock by measuring the dim light melatonin onset (DLMO) as the start of the evening rise of melatonin in plasma or saliva (Lewy and Sack, 1989; Lewy, 1999; Arendt, 2005). It is used as a marker of circadian phase, being useful for determining whether an individual is entrained or not, and for assessing phase delays or advances of circadian rhythms (Pandi-Perumal et al., 2007). At the population level, the DLMO varies with age being earliest in children and latest at the end of adolescence (Kennaway, 2023) and its variability is wider in urban environments than in natural ones (Wright et al., 2013; Moreno et al., 2015). DLMO can also be an accurate proxy of the daily resetting of the circadian phase (Wright et al., 2013; Stothard et al., 2017; Zerbini et al., 2021) and it has long been used to monitor treatments to correct the timing of sleep in the delayed sleep–wake phase disorder (Rahman et al., 2009).

The DLMO itself is affected by circadian entrainers. It is highly sensitive to light, it can be advanced by bright light exposure in the morning (during the circadian phase-advance time window) (Hashimoto et al., 1997; Khalsa et al., 2003; Takasu et al., 2006; Kripke et al., 2007; Kozaki et al., 2016), or delayed by light exposure in the evening (during the circadian phase-delay time window) (Akacem et al., 2018). In addition, individual differences in light sensitivity (Phillips et al., 2019) and photic history either in the short (Stothard et al., 2017) or in the long term (Adamsson et al., 2017) can influence the individual physiological response to light and therefore contribute to individual differences in DLMO. Furthermore, programmed exercise in constant darkness during the morning and evening can also induce DLMO advances and delays, respectively (Youngstedt et al., 2019). Additionally, the melatonin peak is later in healthy nocturnal shift workers than in day-shift ones (Razavi et al., 2019), and the timing of nutrient income has been recognized as an important entrainer of the circadian phase as well (McHill et al., 2019).

The difference between the phase of the circadian oscillator and the phase of the zeitgeber is called phase of entrainment (Pittendrigh and Daan, 1976; Moreno et al., 2020). Inter-individual variations in this phase of entrainment are known as chronotypes and can be estimated from the midpoint of sleep on free days corrected for the sleep debt accumulated over the workweek (MSFsc, Roenneberg et al., 2004; Roenneberg et al., 2019a). Chronotype can also be addressed by scoring self-reported individual preferences for activity and performance (MEQ, Horne and Ostberg, 1976). Both estimations of chronotype have also been used as proxies of the individual circadian phase, with well documented validations of their association to DLMO (Kantermann et al., 2015; Kennaway, 2023).

While theoretical models predict causes and consequences (Asgari-Targhi and Klerman, 2019), and epidemiological studies give statistical support for the design of interventions and public policies (Chen, 2019), realistic scenarios are difficult to approach. Studying real life situations, in which the circadian system is challenged by important alterations in entraining signals, is helpful in exploring its functioning and plasticity. We present the contribution of two natural chronobiological experiments. On one hand, the huge ambient light challenge that a transient trip to the Antarctic summer implies for the biological clock. On the other hand, the model of dancers trained either in early morning or night shifts that allows to evaluate the impact of programmed exercise on circadian and sleep patterns as a particular case of work shifts. These studies are based on two very uncommon ecological situations and involve rather small population samples. Yet, the results obtained from these experiments transcend the uniqueness of these models and show some robust and independent modulations of light and exercise, which are very difficult to observe in realistic scenarios. Most importantly, both studies reinforce the importance of individual characteristics and history. Each person’s lifestyle, including personal environmental, social, and behavioral histories, modulates daily plasticity of the circadian phase and has a predictive power on the way the circadian system will respond to eventual potent entrainers.

## 2 The environmental challenge of the antarctic summer

**FIGURE 1:**
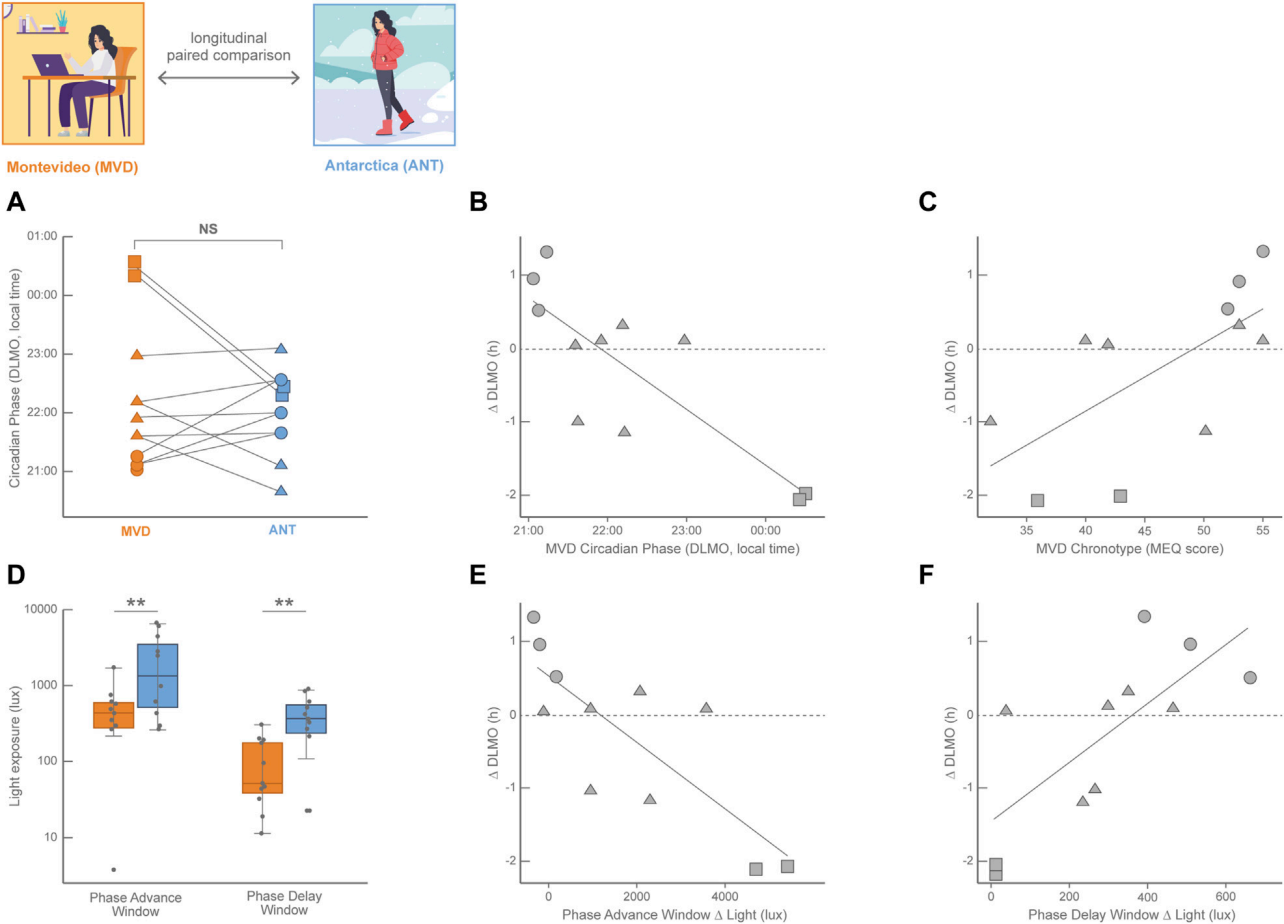
Longitudinal paired comparisons of the circadian phase (DLMO, calculated from 18:00–24:00 saliva samples) and its modulation by light history with data obtained in the fall equinox in Montevideo (orange, MVD, baseline in March 2016) and in the Antarctic summer (blue, ANT, 10-day trip in January 2016) in 11 Uruguayan university students (8 females) with age ranging from 21 to 25 years old. **(A)** Individual changes in the DLMO between both locations; NS: non-significant. Circle symbols correspond to baseline early DLMO (before 21:30); squares correspond to late baseline DLMO (after 00:00) and triangles correspond to intermediate baseline DLMO (from 21:30 to 00:00). Lines connect the values of each participant. **(B)** Pearson linear correlation for the individual circadian phase shift (ΔDLMO, calculated for each participant as DLMO_ANT_ − DLMO_MVD_) with the baseline circadian phase (DLMO in MVD; *R* ^2^ = 0.67, *p* = 0.0019). **(C)** Pearson linear correlation for the individual circadian phase shift (∆DLMO) with the baseline chronotype (Morningness-Eveningness score in MVD, Adan and Almirall, 1990) **(** *R* ^2^ = 0.44, *p* = 0.026). **(D)** Light exposure (obtained from actigraphy data in lux) plotted on a log scale for the phase advance window and phase delay window in both locations. Light exposure was significantly higher in ANT than in MVD in both the phase advance window (Wilcoxon singed-rank test, *p* = 0.019) and the phase delay window (Wilcoxon singed-tank test, *p* = 0.001). **(E)** Pearson linear correlation for the individual circadian phase shift (∆DLMO) between ANT and MVD with the individual difference in light exposure in the phase advance window between ANT and MVD (Phase Advance Window Δ light = light exposure PAW_ANT_ − light exposure PAW_MVD_; R2 = 0.64, *p* = 0.003). **(F)** Pearson linear correlation for the individual circadian phase shift (∆DLMO) between ANT and MVD with the individual difference in light exposure in the phase delay window between ANT and MVD (Phase Delay Window Δ light = light exposure PDW_ANT_ − light exposure PDW_MVD_ R2 = 0.56, *p* = 0.008). The three most early-oriented participants are represented by circles, the two most evening-oriented participants are represented by squares, and intermediate participants are represented by triangles. Data from Silva et al. (2019); Castillo et al. (2023).

Although distorted by electric light and variable across latitudes and seasons, light exposure still impacts on the human circadian system in normal life, leading to light-dependent phase-shifts and changes in sleep patterns (St Hilaire et al., 2012). The Antarctic summer with its 20–24 h of light and 0–4 h of dark and the strict schedule of military bases represents a circadian synchronization condition for the biological clock for both crew residents and visitors. Despite the longer sunlight of summertime impacts in both the phase-advance and the phase-delay windows, several studies have shown the supremacy of the synchronizing power of the morning summer light (Arendt, 2012; Arendt and Middleton, 2018; Castillo et al., 2023). These studies report that circadian rhythms and sleep of the crew of Antarctic bases are usually delayed or even completely desynchronized in the winter with respect to the summer. However, the impact of the Antarctic summer light in visitors during short stays is more difficult to predict and one of the main problems has been the selection of the proper control condition to compare with, which in most cases is their normal lives in their home cities before or after the Antarctic summer trip (Tassino et al., 2016; Moraes et al., 2020). Paradoxically, this normal life implies a less controlled and more variable condition among the participants than the Antarctic summer trip, in which all participants shared the same schedule of activities and meals. Eleven undergraduate students participated of the Uruguayan Summer School on Introduction to Antarctic Research (Facultad de Ciencias, Universidad de la República, Uruguay) that took place during January 17–27, 2016, in the Antarctic Scientific Base Artigas (King George Island, 62°11′ S; 58°52′ W; LD 20:4; Silva et al., 2019; Castillo et al., 2023). The circadian phase (estimated by DLMO) at the end of the Antarctic summer trip was compared to the DLMO measured around the fall equinox in Montevideo (March 7–17, 2016; 34°54′ S; 56°11′ W; LD 12:12; Figure 1; for more details on the study protocols, please refer to (Castillo et al., 2023). DLMO was not significantly different between Montevideo and Antarctica, although less dispersed in Antarctica than in Montevideo (Castillo et al., 2023). Indeed, as shown in Figure 1A, four students displayed an advance in their circadian phase in Antarctica with respect to Montevideo, while seven of them delayed their circadian phase. Interestingly, these irregular phase shifts were not random at all. Two proxies of the participants’ baseline circadian phase; i.e., the DLMO in Montevideo (Figure 1B (Castillo et al., 2023); and the MEQ score in Montevideo (Figure 1C; Silva et al., 2019), had predictive power on the magnitude and valence of the circadian phase shifts. This chronotype-dependent circadian phase shift had consistent behavioral consequences on sleep; participants who advanced their sleep timing in Antarctica respect to Montevideo also advanced their DLMO in Antarctica, while participants who delayed their sleep timing in Antarctica respect to Montevideo also delayed their DLMO in Antarctica (Castillo et al., 2023).

The Antarctic summer means a massive increase of light exposure with respect to the baseline situation in Montevideo around the fall equinox. However, given the wide variation in DLMO observed in Montevideo and the irregular pattern of circadian phase shifts among participants, the impact of light was considered individually during both the phase-advance window (3 h before the DLMO antipode) and the phase-delay window (3 h before the DLMO). As shown in Figure 1D, participants were on average significantly more exposed to light in Antarctica than in Montevideo during both individual light-sensitive windows (Castillo et al., 2023). This seems consistent with the lack of an average DLMO shift, as this great increase in light exposure in both windows with opposite shift effects might be canceling each other and preventing the emergence of a net effect on the circadian phase. Interestingly, this is not the case; rather, shifts of the circadian phase depended on the individual long-term light history (Castillo et al., 2023). The DLMO was advanced in participants exposed to more light in the phase-advance window in Antarctica (Figure 1E) and delayed in participants exposed to more light in the phase-delay window in Antarctica (Figure 1F). This individual impact of the Antarctic trip becomes more comprehensive following the individual cases highlighted in Figure 1. The most early-oriented participants of this study (circles in Figure 1), who showed early baseline DLMOs and high MEQ scores, were much more exposed to light in the Antarctic phase-delay window but not in the phase-advance one. Therefore, these participants delayed their circadian phase in Antarctica with respect to Montevideo, which makes sense for early-oriented individuals entrained by high morning light exposures in their baseline lives. In contrast, evening-oriented participants of this study (squares in Figure 1), who showed late baseline DLMOs and low MEQ scores, were much more exposed to light in the Antarctic phase-advance window but not in the phase-delay one. These participants thus advanced their circadian phase in Antarctica, which makes sense for late-oriented individuals who were most probably sleeping during the morning but active at nighttime in their baseline lives. Participants with intermediate chronotypes and DLMOs (triangles in Figure 1) also support this comprehensive view as they had smaller phase shifts between Montevideo and Antarctica, and thus represent the cases in which the trade-off between the two opposite light influences resulted in a rather small circadian phase shift.

## 3 The challenge of exercise training timing

**FIGURE 2:**
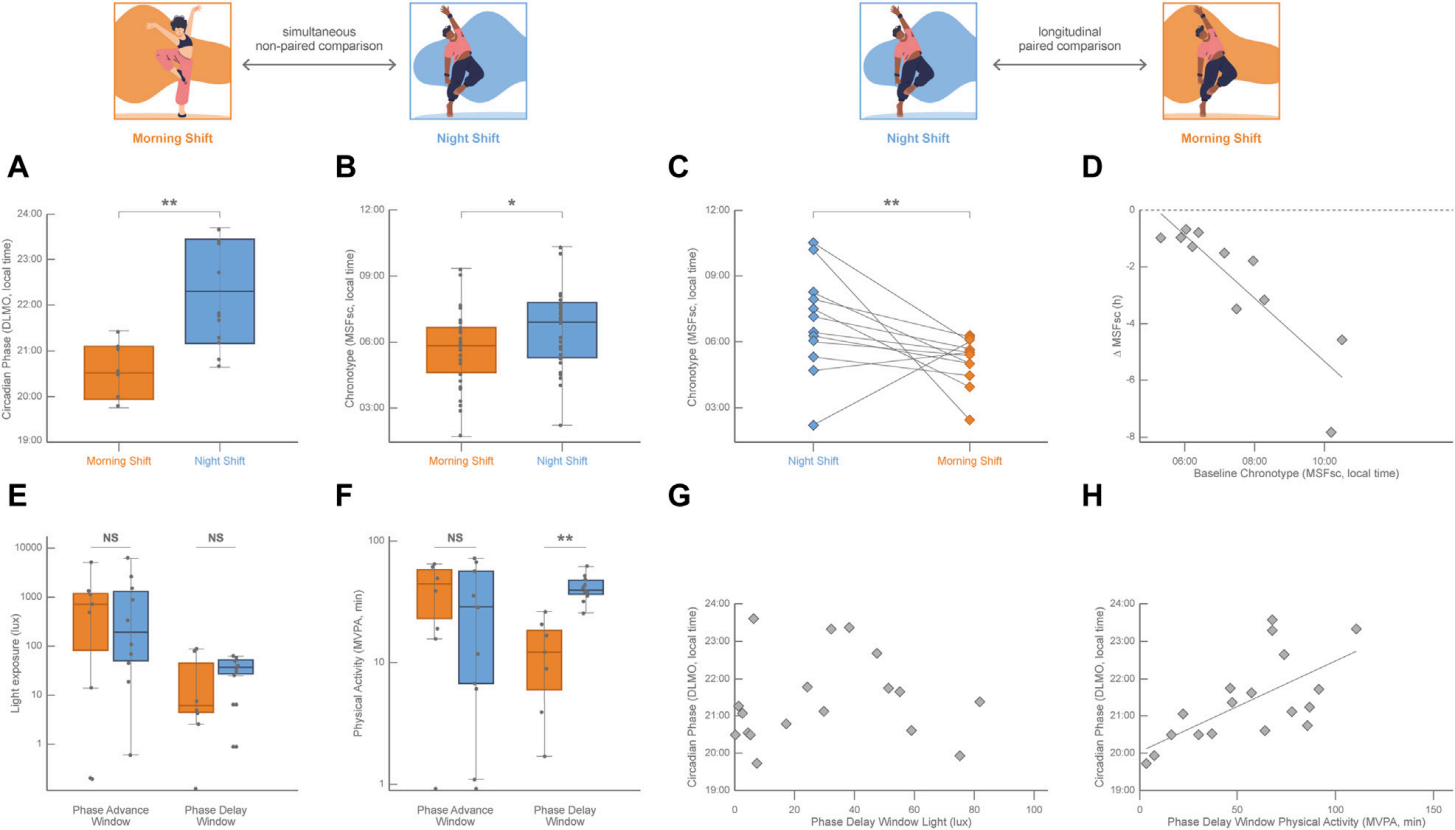
Simultaneous cross-sectional comparisons of the circadian phase (DLMO, calculated from 18:00–24:00 saliva samples) and of the chronotype (MSFsc (Roenneberg et al., 2003); as well as longitudinal paired comparisons of the chronotype between dancers (age ranging from 18 to 30 years old) being trained in the morning shift (orange, 08:30–12:30) and dancers being trained in the night shift (blue, 20:00–24:00) at the Uruguayan public school for professional training in contemporary and folkloric dance. **(A)** DLMO was significantly earlier in dancers of the morning shift (n = 7) with respect to night-shift dancers (n = 11; unpaired Student’s t-test; *p* = 0.005). Data from Coirolo et al. (2022); n = 18 (17 females). **(B)** MSFsc was significantly earlier in dancers of the morning shift (n = 29) with respect to night-shift dancers (n = 27; Mann–Whitney *U* test; *p* = 0.047). Data from Coirolo et al. (2020); n = 56, 45 females. **(C)** MSFsc was significantly earlier in dancers after shifting from the night to the morning shift (n = 12, 10 females; Wilcoxon paired test; *p* = 0.043). Lines connect the values of each participant. Data from Marchesano et al. (2023). **(D)** Pearson linear correlation between the individual chronotype shift (∆MSFsc) and the initial chronotype (MSFsc of dancers attending the night shift; n = 17; *R* ^2^ = 0.79, *p* = 0.0014). Data from Marchesano et al. (2023). **(E)** Light exposure (obtained from actigraphy data in lux) plotted on a log scale for the phase advance window and phase delay window in both morning and night-shift dancers. Light exposure was not significantly different across shifts in both the phase advance window (n = 18; unpaired Student’s t-test, *p* = 0.944) and the phase delay window (n = 18; unpaired Student’s t-test, *p* = 0.564) Data fromCoirolo et al. (2022). **(F)** Time in moderate-vigorous physical activity (physical activity above 100 mg according to Hildebrand et al., 2014; Okely et al., 2018) was obtained from actigraphy data in minutes and plotted on a log scale for the phase advance window and phase delay window in both morning and night-shift dancers. The time spent in moderate-vigorous physical activity was not significantly different across shifts in the phase advance window (n = 18; unpaired Student’s t-test, *p* = 0.579) but was significantly higher in night-shift dancers than in morning-shift dancers in the phase delay window (n = 18; unpaired Student’s t-test, *p* < 0.001). Data from Coirolo et al. (2022). **(G)** No Pearson linear correlation was observed (n = 18; *p* = 0.745) between the individual circadian phase (DLMO) and the individual light exposure in the phase delay window. Data from Coirolo et al. (2022). **(H)** Pearson linear correlation between the individual circadian phase (DLMO) and the time spent in moderate-vigorous physical activity during the phase-delay window (n = 18; *R* ^2^ = 0.42, *p* = 0.003). Data from Coirolo et al. (2022).

While the daily light–dark cycle is the main entrainer of the circadian phase, non-photic inputs like temperature, food availability, social factors, and exercise are also capable of resetting the human circadian phase (Mistlberger and Skene, 2005). Studies in humans under controlled light conditions have identified that the practice of physical activity in the morning induces a circadian phase advance and that physical activity during the evening provokes a circadian phase delay (Barger et al., 2004; Youngstedt et al., 2019; Thomas et al., 2020). While there is abundant evidence of the effects of exercise on circadian phase-shifting in humans under light-controlled conditions (Lewis et al., 2018), it has been very difficult to evaluate the impact of non-photic entrainers in real-life conditions given the compelling evidence of a nonlinear, although partially additive, integration of the influences of the photic and non-photic cues on the circadian phase (Challet and Pévet, 2003; Youngstedt et al., 2019). The Uruguayan national dance college training program (ENFA-SODRE) is a real-life laboratory to study the interplay among biological rhythms, social pressure, and exercise. It operates from Monday to Friday in two extreme shifts scheduled in the morning (from 08:30 to 12:30) and in the night (from 20:00 to 24:00; for more details on the study protocols, please refer to (Coirolo et al., 2020; Coirolo et al., 2022; Marchesano et al., 2023). Several ecological conditions of this paradigm make it especially advantageous (Coirolo et al., 2020; Coirolo et al., 2022): a) dance students were not able to choose their training shift; b) although first and second-grade dance students attended the night shift while dance students of the third and fourth grade attended the morning shift, the age of participants was not different between shifts; c) when recorded around the middle of the school year during the austral winter (August 10–27, 2019), dancers of both training shifts had an adequate weekly average sleep duration (above 7 h per day) and showed the expected pattern of delaying their sleep in weekends with respect to training days; d) actimetry recording showed that dancers of the morning and night shifts were exposed to a similar daily intensity of light exposure; and that e) morning and night-shift dancers displayed a similar daily physical activity with also similarly robust activity-rest circadian rhythms. However, dancers of the morning shift showed earlier circadian phases than night shift dancers, which was 1.5 h earlier in average estimated by DLMO (measured at the end of the recording period; Figure 2A; Coirolo et al., 2022), and 1 h earlier in average estimated by MSFsc (measured at the beginning of the recording period; Figure 2B; Coirolo et al., 2020). Sleep timing was also earlier on training days and sleep duration shorter on weekends in morning-shift dancers than in night-shift dancers (Coirolo et al., 2022; Estevan et al., 2023). Interestingly, dancers of the night shift who were followed up 2 years later, when they were trained in the morning shift, showed a significant advance in their circadian phase estimated by MSFsc (reported on 3 September 2021, Figure 2C) and earlier sleep timing on both training and free days (Marchesano et al., 2023). Even more important, this paired longitudinal chronotype advance was associated with their initial circadian phase (estimated by MSFsc, Figure 2D), reinforcing at the same time the huge plastic capacity of the circadian system and the importance of the predictive value of the initial indicators of the circadian phase against future social demands.

Although dance training meant a similar physical challenge for dancers of both shifts, the timing of physical activity was obviously different across shifts (Coirolo et al., 2022). Accordingly, daily light exposure intensity was also similar for dancers of both shifts, while its timing was not (Coirolo et al., 2022). However, the expected association between physical activity and light exposure patterns was not observed in these dancers. For example, morning-shift dancers displayed more intense physical activity than night-shift dancers but were exposed to a similar light intensity during the time of the morning training shift. This incongruence between light and exercise patterns was exploited to unravel potential confounding effects. Given the highly diverse individual internal timing among dancers (4 h variation of the circadian phase estimated by DLMO), the effects of light and physical activity were analyzed during each participant’s phase-sensitive time windows. At the phase-advance window (in the antipode of the DLMO), light exposure (Figure 2E) and the time spent in displaying moderate-vigorous physical activity (Figure 2F) were not different across shifts, and neither correlated with DLMO. During the phase-delay window (around the DLMO) all dancers were similarly exposed to a low light intensity (Figure 2E) that did not correlate with DLMO (Figure 2G); while the time spent in displaying moderate-vigorous physical activity was higher in night-shift dancers than in morning shift dancers (Figure 2F) and positively correlated with DLMO (Figure 2H). In this real-life experiment of dancers being trained in shifts, it was possible to detect that the amount of physical activity displayed during dancers’ phase-delay window was correlated to later dancers’ circadian phase, whereas light exposure was not. Even though photic stimuli are the most potent entrainers of the circadian clock, this is an example of the phase-delay effect of exercise in real-life conditions in a peculiar natural model system in which environmental light is not associated with the modulation of the circadian phase.

The lack of influence of light in modulating the circadian phase of these dancers trained in shifts should in no way call into question the universal effect of light as a potent entrainer of circadian rhythms including the sleep-wake cycle. The analysis of daily sleep patterns estimated from accelerometry in the same group of dancers showed an influence of morning light exposure on advancing sleep onset and increasing sleep duration; as well as an influence of late evening light exposure on delaying sleep (Estevan et al., 2023). In the same sense, the lack of effect of morning regular physical activity in advancing the circadian phase in this natural model system should not undermine the evidence that indicates its protective role on sleep and performance (McMorris, 2021) In this same group of dancers, dance training in the morning improved cognitive and motor performance at noon, while attentional performance was positively associated with the amount of exercise time in the morning (Marchesano, 2022).

## 4 Discussion

Neuroethologists have coined the expression “champion species” to refer to species that makes greatest use of one feature or ability and thus optimize the study of its underlying mechanisms (Heiligenberg, 1991). Inspired by this principle and by some previous studies in real-life conditions (Wright et al., 2013; Stothard et al., 2017), we revised two natural human chronobiology experiments that represent “champion conditions” in which the circadian system is extremely challenged in its operation. Both experiments have in common a longitudinal approach in which plastic changes in the circadian phase are demonstrated in response to a complex environmental-behavioral-social challenge. When the challenge is socio-environmental (trip to the Antarctic summer), individual light history is associated with the observed circadian phase shifts. When the challenge is mostly socio-behavioral (dancers trained in extreme shifts), the training schedule imposes a major influence on the circadian phase and in sleep patterns, while programmed exercise adds its influence to delay the circadian phase when it is practiced at night. These “champion models” are also key to evaluate the multiple dynamic interactions between photic and non-photic stimuli (food intake, temperature, arousal, stress, exercise; Bass and Takahashi, 2010; Hamaguchi et al., 2015; Tahara et al., 2017) for the daily adjustment of the circadian phase in realistic scenarios. For example, it would be interesting to evaluate how regular Antarctic schedules of awake time and meals interact with light in the modulation of the individual circadian phase. It would also be exciting to further explore the interactions between light and exercise in dancers trained in shifts, whose circadian phase seems independent of evening light while sleep timing is not (Estevan et al., 2023).

Both experiments show that changes of the circadian phase in response to interacting entrainers are diverse among individuals and depend on the individual baseline circadian phase. This is very important and promising to adjust potential light, exercise, or pharmacological interventions (Ruben et al., 2019; Vetter et al., 2022; Klerman et al., 2023). Interestingly, both experiments also show that the baseline circadian phase is highly variable among individuals with DLMO range of around 4 h in both cases. This reinforces the general conception that urban life increases circadian misalignment, meaning that activities set at a given local time, which is the time that governs our social agenda, can be very different from each individual circadian time, which is the time that endogenously governs physiology and behavior. Any action or intervention cannot disregard this diversity and shall be tailored individually. Since going back in time to be highly and homogeneously synchronized with the natural light-dark cycle is not an option for the current human population, chronobiological evaluations, and the potential interventions that derive from them, must start from the consideration of this basal diversity. Average diagnoses and general recommendations are of course a good start point while individual multimodal assessment is still not feasible. However, in real-life scenarios of extreme misalignment, as the examples revised in this article, individual assessment becomes crucial for accurate diagnosis and adequate treatment of circadian disorders that affect health and wellbeing.

## Data Availability

The data analyzed in this study is subject to the following licenses/restrictions: Datasets of the original articles that were used in this perspective article are available upon request to the corresponding author. Requests to access these datasets should be directed to AS, asilva@fcien.edu.uy BT, tassino@fcien.edu.uy.
